# Quantitative microbial faecal source tracking with sampling guided by hydrological catchment dynamics

**DOI:** 10.1111/j.1462-2920.2008.01682.x

**Published:** 2008-10

**Authors:** G H Reischer, J M Haider, R Sommer, H Stadler, K M Keiblinger, R Hornek, W Zerobin, R L Mach, A H Farnleitner

**Affiliations:** 1 Institute of Chemical Engineering, Gene Technology Group, Vienna University of TechnologyGetreidemarkt 9/166-5-2, A-1060 Vienna, Austria; 2 Clinical Institute of Hygiene and Medical Microbiology, Medical University ViennaKinderspitalgasse 15, A-1090 Vienna, Austria; 3 Institute of Water Resources Management, Hydrogeology and Geophysics, Joanneum ResearchElisabethstraße 16/II, A-8010 Graz, Austria; 4 Institute for Water Quality and Waste Management, Department for Chemistry and Biology of Water, Vienna University of TechnologyKarlsplatz 13, A-1040 Vienna, Austria; 5 Vienna WaterworksGrabnergasse 4-6, A-1060 Vienna, Austria

## Abstract

The impairment of water quality by faecal pollution is a global public health concern. Microbial source tracking methods help to identify faecal sources but the few recent quantitative microbial source tracking applications disregarded catchment hydrology and pollution dynamics. This quantitative microbial source tracking study, conducted in a large karstic spring catchment potentially influenced by humans and ruminant animals, was based on a tiered sampling approach: a 31-month water quality monitoring (Monitoring) covering seasonal hydrological dynamics and an investigation of flood events (Events) as periods of the strongest pollution. The detection of a ruminant-specific and a human-specific faecal *Bacteroidetes* marker by quantitative real-time PCR was complemented by standard microbiological and on-line hydrological parameters. Both quantitative microbial source tracking markers were detected in spring water during Monitoring and Events, with preponderance of the ruminant-specific marker. Applying multiparametric analysis of all data allowed linking the ruminant-specific marker to general faecal pollution indicators, especially during Events. Up to 80% of the variation of faecal indicator levels during Events could be explained by ruminant-specific marker levels proving the dominance of ruminant faecal sources in the catchment. Furthermore, soil was ruled out as a source of quantitative microbial source tracking markers. This study demonstrates the applicability of quantitative microbial source tracking methods and highlights the prerequisite of considering hydrological catchment dynamics in source tracking study design.

## Introduction

The contamination of water resources by faecal pollution constitutes a significant risk to human as well as animal health because many pathogens are associated with faeces (WHO, 2004). Traditionally the assessment of health-related microbial water quality as required by regulations (Anon, 1998; 2006) is based on the enumeration of faecal indicator bacteria (e.g. *Escherichia coli*, enterococci). These parameters should be indicative for the potential presence of pathogens with faecal origin in a water resource (WHO, 2004). However, they provide no information as to the source of the pollution because indicators might originate from humans (usually waste water), livestock or wild animals. Microbial source tracking methods are supposed to allow the identification of specific faecal source groups. This in turn permits directed, efficient and cost-effective remediation efforts in the catchment like the restriction of livestock numbers or the improvement of waste water collection and treatment. Microbial source tracking can then also be used to evaluate the efficiency of such measures.

Various microbial source tracking methods have been proposed in the past (reviewed in Simpson *et al*., 2002; Seurinck *et al*., 2005a; Field and Samadpour, 2007). Among those, methods for the detection of source-specific genetic markers found in dominant faecal anaerobes (e.g. *Bacteroidetes*, bifidobacteria) by PCR have attracted a lot of attention (Bernhard and Field, 2000a,b; Bonjoch *et al*., 2004) and were shown to be very reliable if compared with other methods (Griffith *et al*., 2003; Noble *et al*., 2006; Gourmelon *et al*., 2007). So far, such markers have been proposed for general faecal pollution as well as for specific sources like human, ruminant, dog, pig or horse faeces (Domingo *et al*., 2007). The methods have been applied in several field studies (Bernhard *et al*., 2003; Boehm *et al*., 2003; Bower *et al*., 2005; Noble *et al*., 2006; Shanks *et al*., 2006; Gourmelon *et al*., 2007). However, problems with specificity, high detection limits and the lack of quantitative data limit the conclusions that can be drawn from application of conventional PCR methods, especially in catchments with multiple faecal source groups. Recently quantitative methods based on real-time PCR (qPCR) were developed (Seurinck *et al*., 2005b; Layton *et al*., 2006; Reischer *et al*., 2006; 2007; Kildare *et al*., 2007; Okabe *et al*., 2007) which exhibit higher specificity. Only two of these methods have been applied in the field in short-term studies (1–2 months), too short to cover seasonal pollution dynamics (Seurinck *et al*., 2006; Savichtcheva *et al*., 2007). Up to date there was a severe lack of integrated study approaches which combine quantitative microbial source tracking with catchment hydrology and traditional monitoring parameters.

The aim of this study was to apply modern quantitative microbial source tracking methods on a large and complex karstic spring catchment in context with hydrology and other water quality parameters over a prolonged period of time in order to comprehensively, qualitatively and quantitatively characterize the pollution sources. To this end, the study was based on three complementary strategies: (i) a 31-month monitoring programme (Monitoring) with fortnightly to monthly sampling to cover the whole seasonal range of hydrological conditions affecting water quality, (ii) an in-depth analysis of periods with strong surface influence during summer flood events (Events), and (iii) a multiparametric statistical analysis of microbiological, hydrological and chemophysical parameters measured in all samples. The human-specific BacH and the ruminant-specific BacR methods, especially developed for this catchment, were used for source determination (Reischer *et al*., 2006; 2007). The study site chosen for this investigation was the large catchment area of the limestone karstic spring LKAS2 which is an important raw water source situated in Austria's Northern Calcareous Alps (cf. *Experimental procedures*). The water quality of this spring is occasionally degraded by faecal contamination from its catchment where human activity and ruminant animals (cattle, deer and chamois) represent the potential sources of faecal contamination (Burtscher *et al*., 2006; Reischer *et al*., 2006; 2007). In addition to spring water quality monitoring, different soils in the catchment area were investigated for their potential as a source of the quantitative microbial source tracking markers which might limit the applicability of the methods. This is the first study integrating catchment dynamics in source tracking study design and demonstrating the potential of quantitative microbial source tracking for quantitative faecal source determination and allocation.

## Results

### Hydrological characteristics of the spring

**Fig. 1:**
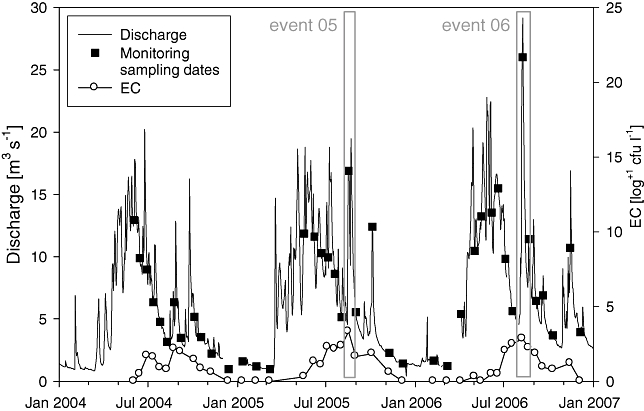
Hydrological situation in the karstic spring LKAS2 during the study period. Discharge levels are daily mean values. Small squares mark the sampling dates during the Monitoring programme, grey boxes outline the Events 05 and 06 sampled more extensively. The gap in the discharge data was due to malfunctions of the instruments necessary for the calculation of the discharge. EC, *E. coli*.

During the study period from 2004 to 2006 the limestone karstic aquifer spring LKAS2 generally showed typical annual fluctuations in spring discharge (Stadler and Strobl, 2006), with snowmelt starting earlier than usual in 2005. In both 2005 and 2006, discharge was marginally higher than the mean discharge between 1995 and 2006, whereas the discharge in 2006 was slightly higher than in 2005. As significant infiltration of water to the aquifer happens during snowmelt and summer, discharge was the highest during spring and summer with flood events caused by snowmelt or heavy precipitation in the catchment area and lower during wintertime (catchment covered in snow). The fortnightly to monthly sampling scheme covered most of the range of hydrological seasonal dynamics during the 31-month study period (Fig. 1). The Events monitored in summers 2005 (Event 05) and 2006 (Event 06) were characteristic late summer flood events, caused by thunderstorms, starting at 5 m^3^ s^−1^ daily mean discharge. Precipitation was 195 mm in Event 05 (14 August to 21 August 2005) with two main events (which caused a double-peak flood event) and 289 mm in Event 06 (3 August to 20 August 2006). The peaks at about 20 m^3^ s^−1^ (Event 05) and 29 m^3^ s^−1^ (Event 06) were among the highest daily mean discharge observed in this spring since hydrological observations started in 1995 (Stadler, 2006).

### Microbiological water quality and faecal pollution status

Microbiological water quality of spring water investigated using faecal indicators (*E. coli*, enterococci, *Clostridium perfringens*) and copiotrophic indicators (HPC, aerobic spore-formers) showed high variation during the seasonal cycles reflecting the general hydrological situation (Table 1) and is exemplarily shown for *E. coli* in Fig. 1. The strongest faecal impact is evident during the summer months where *E. coli* levels reached up to 2.1 × 10^3^ colony-forming units (cfu) l^−1^ during summer flood Events. Generally, microbiological parameters showed much higher median levels in the Event samples than during the Monitoring (Table 1). A general correlation among the microbiological quality parameters was evident (Table 2) with higher correlation coefficients during the Events. In all three data sets a clear correlation was observable between the microbiological indicator parameters *E. coli* (EC), enterococci (ENT), presumptive *C. perfringens* (pCP) and heterotrophic plate count at 22°C (HPC22) (*r* = 0.53–0.91), while aerobic spore formers correlated only in Event situations. The spectral absorbance coefficient at 254 nm (SAC_254_), a measure for organic matter content, was also significantly correlated with the microbiological quality parameters (Table 2).

**Table 1:** Medians and ranges of parameters determined in LKAS2 during the study

Parameterunit		BacR^a^ME l^−1^	BacH^a^ME l^−1^	EC^a^cfu l^−1^	ENT^a^cfu l^−1^	pCP^a^cfu l^−1^	HPC22^a^cfu l^−1^	Aerob^a^cfu l^−1^	Dis^a^m^3^ s^−1^	SAC_254_m^−1^	TurbNTU	CondμS cm^−1^
Monitoring	Median	2.9	< 0.8^b^	0.8	0.7	0.3	1.6	2.9	3.8	1.58	0.14	192
*n* = 42	Range	< 0.8^b^−5.0	< 0.8^b^−3.6	n.d.−3.3	n.d.−2.7	n.d.−1.4	n.d.−3.3	2.4–5.2	3.0–4.2	0.22–7.57	0.03–12.90	154–233
Event 05	Median	4.7	1.3	2.4	1.9	0.7	2.6	3.7	4.2	6.78	1.55	196
*n* = 24	Range	2.8–5.9	< 0.8^b^−3.1	1.7–3.3	1.0–2.8	n.d.−1.5	2.0–3.7	2.6–4.6	3.7–4.3	1.83–9.85	0.31–2.95	190–203
Event 06	Median	4.2	1.1	2.4	2.0	0.7	2.6	3.6	4.1	3.35	2.29	184
*n* = 27	Range	2.6–5.2	< 0.8^b^−2.7	1.6–3.1	0.7–2.5	n.d.−1.7	1.6–2.9	2.7–4.5	3.7–4.6	0.66–6.14	0.23–12.9	175–192

a Data log^+1^ transformed

b log^+1^ of the detection threshold 5 ME l^−1^

BacR, ruminant-specific marker; BacH, human-specific marker; EC, *E. coli*; ENT, enterococci; pCP, presumptive *Clostridium perfringens*; HPC22, heterotrophic plate count at 22°C; Aerob, aerobic spore-formers; Dis, discharge; SAC_254_, spectral absorbance coefficient at 254 nm; Turb, turbidity; Cond, conductivity; ME, marker equivalents; n.d., not detectable.

**Table 2:** Correlation analysis of data collected during Monitoring (*n* = 42), Event 05 (*n* = 24) and Event 06 (*n* = 27)

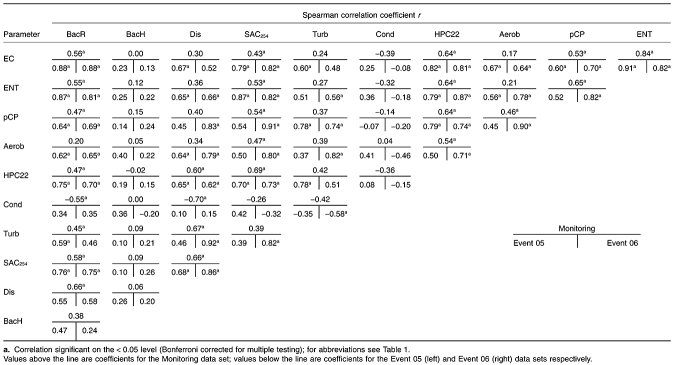

### Quantitative microbial source tracking during Monitoring

**Fig. 2:**
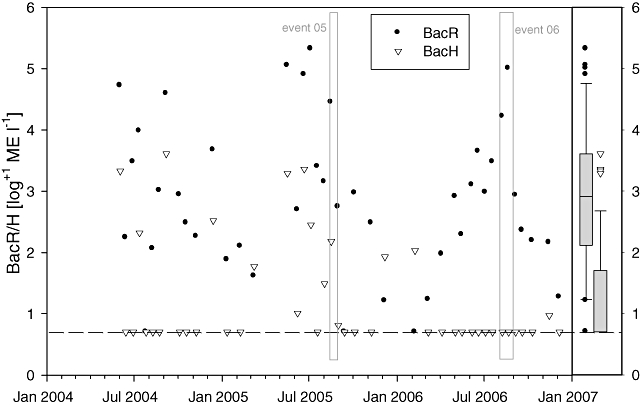
BacH and BacR results for LKAS2 from June 2004 to December 2006. Data are given as marker equivalents (ME) per litre of spring water after log^+1^ transformation; black dots are results for ruminant-specific BacR marker, grey triangles for human-specific BacH marker; box plots on the right show the distribution of the quantitative microbial source tracking marker values (whiskers, 10th and 90th percentile; boxes, 25th and 75th percentiles; lines within the box, median); the dashed line marks the detection threshold with regard to the used filtration and sample DNA volume, all results lying on this line were not detectable in qPCR and consequently had a concentration < 5 ME l^−1^; grey boxes outline the Events 05 and 06.

The ruminant-specific BacR marker was detectable in 40 out of 42 samples (95%) analysed during the basic Monitoring with a median concentration of 8.0 × 10^2^ BacR marker equivalents (ME) l^−1^ (Fig. 2). In contrast the human-specific BacH marker was detectable in 15 (36%) out of 42 samples with a 75th percentile concentration of 5.8 × 10^1^ ME l^−1^ in contrast to a 75th percentile of 4.5 × 10^3^ ME l^−1^ for BacR. Interestingly the BacH marker was occasionally detected in 2004 and 2005, especially during summer months, but was almost consistently not dectable in samples from 2006 (Fig. 2). The concentration of the BacR and BacH markers reflected the strong annual fluctuations of spring discharge with lower numbers in winter and higher numbers in summer (cf. Figs 1 and 2).

### Quantitative microbial source tracking during flood Events

**Fig. 3:**
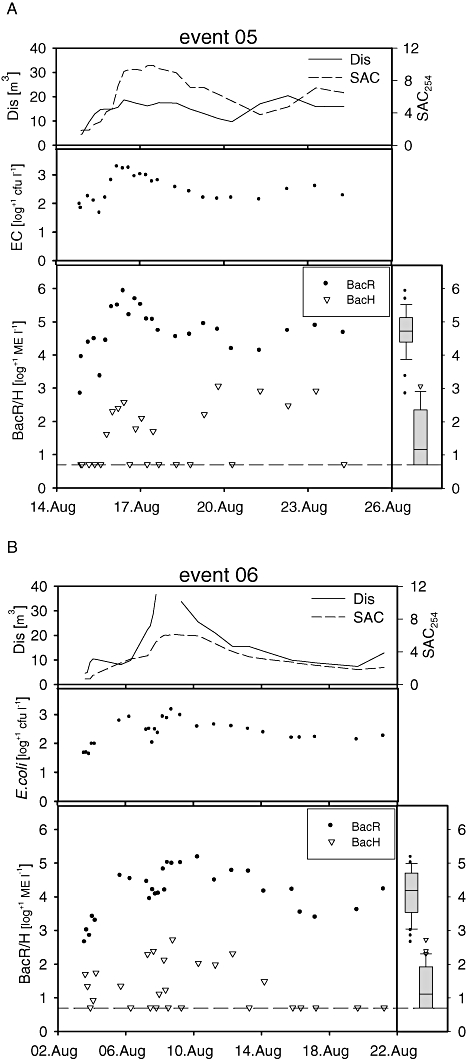
Course of the investigated summer Events 2005 (A) and 2006 (B). Upper parts: discharge and spectral absorption coefficient; middle parts: *E. coli* concentrations in cfu l^−1^ after log^+1^ transformation; lower parts: BacH and BacR results in marker equivalents (ME) per litre after log^+1^ transformation; box plots on the right show the distribution of the quantitative microbial source tracking marker values (whiskers, 10th and 90th percentile; boxes, 25th and 75th percentiles; lines within the box, median); the dashed line marks the detection threshold with regard to the used filtration and sample DNA volume, all results lying on this line were not detectable in qPCR and consequently had a concentration < 5 ME l^−1^; Dis, discharge; SAC_254_, spectral absorbance coefficient at 254 nm; BacH, human-specific marker; BacR, ruminant-specific marker; EC, *E. coli*.

The BacR parameter was detectable throughout the course of the Event 05 at concentrations ranging from 6.7 × 10^2^ ME l^−1^ to 8.2 × 10^5^ ME l^−1^ while the BacH parameter was detected in 50% of the samples with concentrations at least three orders of magnitude lower than the BacR numbers in the same sample (Fig. 3A). Compared with that, concentrations of cultivable *E. coli* ranged from 45 cfu l^−1^ in the first phase of the Event to 1.9 × 10^3^ cfu l^−1^ during the peak.

Event 06 was a particularly strong flood event. A mean daily discharge of 29 m^3^ s^−1^ was registered on 7 August and during the next day there was a discharge data gap caused by the destruction of the instruments needed for determination of discharge by the flood. The BacR marker was again detected in all samples (median 1.5 × 10^4^ BacR ME l^−1^), the BacH marker in 15 out of 27 samples (56%). The faecal indicator levels showed very similar dynamics as in Event 05. For example, cultivable *E. coli* levels ranged from 40 cfu l^−1^ to 1.4 × 10^3^ cfu l^−1^ (Table 1).

### Multiparametric data analysis

**Fig. 4:**
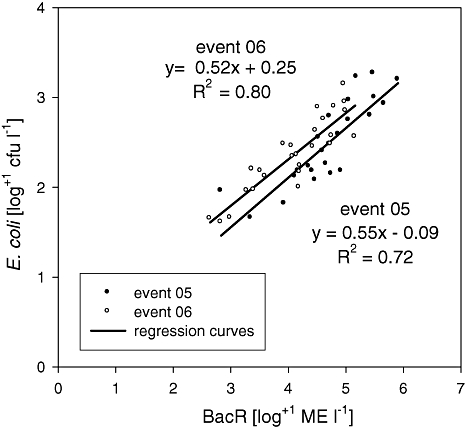
Regression analysis of BacR and *E. coli* data from Events 05 and 06. BacR, ruminant-specific marker; ME, marker equivalents.

To determine whether the quantitative microbial source tracking parameters are quantitatively relatable to other microbiological, chemophysical and hydrological parameters, multiple correlation analysis (Spearman rank correlation coefficients *r*; significance level < 0.05, Bonferroni corrected) was performed for the three data sets Monitoring, Event 05 and Event 06 (Table 2). The BacR results were highly correlated with the standard faecal indicators, especially EC and ENT, and to some lesser extent with pCP, HPC22 and SAC_254_. The highest coefficients were evident between BacR and EC during the Events (*r* = 0.88 in both). Correlations between these two parameters were evident but less pronounced during the Monitoring. In contrast, the BacH marker concentrations showed low, non-significant correlations with other parameters during the Monitoring as well as the Events (Table 2). Regression analysis of BacR and EC during the Events was performed to investigate the relationship between those parameters in more detail. The regression curves of the two Events were remarkably similar, with coefficients of determination *R*^2^ of 0.72 and 0.80 for Event 05 and Event 06 respectively (Fig. 4).

### Occurrence of quantitative microbial source tracking markers in soil of the catchment

To investigate soil as a possible source or storage reservoir for the quantitative microbial source tracking markers, 130 soil samples were collected throughout the study area. These samples comprised 82 soil samples randomly collected throughout the catchment area and soil samples from three ecologically defined type regions (summer pastures, woodland, alpine sward; *n* = 48) typical for the catchment area. The BacH marker was not detectable in any soil samples. The BacR marker was only detected in nine soil samples (6.4%), seven of those originating from mountain pastures with substantial cattle populations (> 100 animals) as immediate sources of ruminant faecal input.

## Discussion

### Requirements for modern microbial source tracking methods

Recently, a basic set of goals on the road to modern microbial source tracking methods has been defined by Domingo and colleagues (2007). Among these are a high level of specificity and sensitivity for the respective faecal source group, a basic performance criterion that always should be investigated before applying a microbial source tracking method in a new study area. The BacH and BacR methods were developed during 2005 and 2006 concurrently with the present study (2004–2006). Their specificity, sensitivity, host distribution and abundance were successfully evaluated using faecal samples (*n* > 360) from the same study area (Reischer *et al*., 2006; 2007). They meet the often voiced demand for the use of quantitative methods necessary to compare quantitative microbial source tracking data with other water quality parameters. The detection of at least one of the markers in all but two water samples tested in this study demonstrates the environmental occurrence and temporal genetic stability of the used quantitative microbial source tracking markers as well as the robustness and sensitivity of the detection method.

When conducting microbial source tracking investigations, special attention should also be given to study design as the adaption of sampling schedules and frequencies to the prevalent hydrological conditions and pollution dynamics are crucial for the significance of the results (Domingo *et al*., 2007; Kay *et al*., 2007). The present study established and evaluated a new sampling concept with consideration for the whole seasonal hydrological catchment variability and special emphasis on strong pollution events. It was conducted in two tiers: first, a Monitoring programme to give insight into the levels of quantitative microbial source tracking markers as well as other microbiological and chemophysical parameters under seasonally strongly differing hydrological conditions; second, the investigation of summer flood Events at a much higher time resolution to provide detailed insight during the periods with the highest surface input and matter transfer in the aquifer. Hydrological events have been shown to account for > 90% of the catchment-derived faecal indicator flux from diffuse source pollution (Lee *et al*., 2002) and are consequently the most relevant periods for the management of water resources.

### Tiered sampling approach for quantitative microbial source tracking in the karstic spring LKAS2

The tiered approach applied in this quantitative microbial source tracking study allowed the unambiguous identification of ruminant animal faecal sources as the main origin of faecal contamination in the catchment of LKAS2. The BacR marker was detected in higher frequency and at levels far exceeding the human-specific BacH marker numbers in all phases of the study (overall ratio of medians BacR/BacH = 2 × 10^3^). The dominance of ruminant markers was even more pronounced when considering that median BacR marker concentrations in ruminant faeces (4.0 × 10^9^ BacR ME g^−1^ faeces) (Reischer *et al*., 2006) are around one order of magnitude lower than median BacH numbers in human faeces (3.2 × 10^10^ BacH ME g^−1^ faeces) (Reischer *et al*., 2007). The fact that the BacH marker was occasionally detected in the summers of 2004 and 2005 but never during most of 2006 might be related to the replacement of an old mountain hut in the autumn of 2005. The hut received large numbers of visitors every summer, was one of the potentially important sources of human faecal pollution in the catchment and lacked proper sewage disposal. Since 2006 faeces from the new hut have been transported to a treatment plant outside the catchment. In contrast to the Monitoring data, the BacH marker was detected during Event 06. This discrepancy might be due to the enormous nature of this Event where precipitation was heavy throughout the whole catchment possibly mobilizing human faecal sources usually not contributing to the aquifer.

It should be highlighted that the potential faecal sources in our study area are dominated by humans and ruminant animals. Public records show that only ruminant livestock is present in the catchment while regular inspections of the catchment area by gamekeepers and employees of the waterworks show that the wild animal population consists mainly of the ruminants chamois, deer and roe deer. However, the applicability of the tiered approach is not limited to source tracking studies with only two potential source groups. This comprehensive concept should be very suitable for catchments influenced by multiple faecal sources (including birds or non-ruminant livestock), provided that molecular diagnostic capabilities support discrimination of targeted faecal materials and respective host groups.

### Multiparametric analysis: linking quantitative microbial source tracking and standard faecal indicators

Karstic springs are the source for approximately 50% of the raw drinking water needed for Austria's water supply. Their occasional vulnerability to surface influence makes them interesting targets for the conduction of microbial source tracking studies. In order to close the gap between microbial source tracking methods and standard water quality parameters the present study embedded quantitative microbial source tracking data in a larger set of microbiological, chemophysical and hydrological data. The regression analysis between BacR and *E. coli* during the flood Events demonstrates that 72% (Event 05) and 80% (Event 06) of the variation in *E. coli* was attributable to the variation in BacR levels. This is the first study to demonstrate the ability of quantitative microbial source tracking studies to quantitatively link source-specific marker levels to general faecal pollution indicators in order to estimate the contribution of one source group to total faecal pollution as measured in conventional faecal monitoring. The strong resemblance of the regressions from the two Events in terms of slope and position is further indication of the strong coherence between BacR and *E. coli* (cf. Fig. 4).

It is interesting to note that correlation coefficients, especially those between the BacR parameter and the standard faecal indicators (e.g. *E. coli*), were generally higher in the flood Event data sets than in Monitoring data (cf. Table 2). This fact can be explained by increased transfer rates of surface-associated materials (including faecal material) to the spring outlet at times of strong precipitation, enhanced surface run-off and resulting flood events in the aquifer. Transfer times during such an event are often as low as a few hours in LKAS2. For the application of quantitative microbial source tracking methods this means that differential persistence of quantitative microbial source tracking markers and standard faecal indicators does not have a strong influence on the relation between those parameters during events with very short hydrological retention times. Consequently event scenarios appear highly suitable for linking quantitative microbial source tracking parameters with standard faecal indicators. On the other hand, at periods with higher retention times (predominantly in winter) differential environmental persistence plays a larger role which might explain the lower but nonetheless significant correlations during the Monitoring. *Bacteroidetes*-based quantitative microbial source tracking markers have been shown to be more persistent than faecal indicators like *E. coli* at the low water temperature (Seurinck *et al*., 2005b; Okabe and Shimazu, 2007) prevalent in the studied karstic spring (median water temperature 5.2°C). Tests of quantitative microbial source tracking marker and standard faecal indicator persistence in faecal suspensions prepared with LKAS2 spring water support these findings (data not shown).

When investigating soil from the catchment, the BacR marker was only detected in areas with direct faecal influence whereas the BacH marker was never detected. This allows the conclusion that soil in the study area is not a source of the *Bacteroidetes* populations detected with the quantitative microbial source tracking assays while it might serve as a transient storage reservoir. Previous studies in the catchment showed that the numbers of faecal indicator organisms in soil alone were too low to account for the concentrations found in the spring water during flood events (Burtscher, 2001). The conclusion that the common source for the BacR markers and standard faecal indicators in the spring is indeed ruminant faeces and not soil or other sources is also supported by the relatively low correlation between the marker levels and the numbers of copiotrophic HPC and aerobic spore-formers (cf. Table 2). The aerobic spore-former parameter has previously been shown to be indicative for soil input rather than input from faecal material (Fujioka, 2001).

This study showed that the thorough investigation of catchment hydrology and pollution dynamics is a prerequisite for successful quantitative microbial source tracking study design. Long-term Monitoring of water quality provided insight into seasonal quantitative microbial source tracking dynamics. The multiparametric analysis of hydrological Events allowed the quantitative allocation of a large part of general faecal pollution (as determined by standard faecal indicators) to the ruminant source group. By applying modern quantitative microbial source tracking methods in this manner, future studies might be able to achieve source identification and quantitative allocation in complex catchments with multiple faecal source groups.

## Experimental procedures

### Study area

The studied alpine karst system is located in the so-called Northern Calcareous Alps in Austria reaching altitudes up to approximately 2300 m above sea level (a.s.l.) (47°35′−47°43′N, 15°−15°20′E). The selected alpine spring is directly accessible in a valley at 600 m a.s.l. (Stadler and Strobl, 1997; 2001). Limestone karstic aquifer spring 2 (LKAS2) is a typical limestone spring type according to D'Amore and colleagues (1983) having well-developed karst conduits (Stadler and Strobl, 1997). The mean discharge was 5145 l s^−1^ (1995–2006) showing high variations with a discharge_max_/discharge_min_ ratio of ∼82 based on daily mean discharges. The mean water residence time was estimated between 0.8 and 1.5 years and the discharge response after precipitation is very quick (2–12 h). The estimated alpine catchment area is about 70 km^2^ in size with a mean altitude of 1380 m a.s.l. (Stadler and Strobl, 2001). Vegetation comprises summer pastures, natural calcareous alpine swards with open krummholz and forests (Dirnböck *et al*., 1999).

### Hydrological and chemophysical data

All hydrological and chemophysical data were recovered by in-field on-line sensors directly installed at the spring outlet of LKAS2. Conductivity- and discharge-related parameters (water pressure, current meters, inductive discharge measurements) were registered with the data-collecting system GEALOG-S from Logotronic (Vienna, Austria). Probes used for measuring conductivity and water pressure were WTW-Tetracon 96A (WTW, Weilheim, Germany) and PDCR 1830 (Druck, London, UK) respectively. Signals from these sensors were converted with discharge stage relations (Stadler and Strobl, 2000). Data were stored every 15 min which comprised the data basis for all hydrographical investigations. All sensors were controlled with single measurements with an interval of 1–4 weeks, using instruments, which were part of a certificated quality management system. Turbidity and SAC_254_ were measured with a spectro::lyser (scan Measuring Systems, Vienna, Austria).

### Water sampling and sample processing

Water samples were taken from LKAS2 between June 2004 and December 2006 with fortnightly sampling in summer (May to September) and monthly sampling during the rest of the year (Monitoring, *n* = 42). Additionally, two Events during high discharge conditions after heavy precipitation were sampled in August 2005 (Event 05; 17-day period; *n* = 24) and in August 2006 (Event 06; 18-day period; *n* = 27) with a higher time resolution (intervals between 1 h and 48 h). Sampling for the Monitoring was continued independently during the Event sampling.

Water samples were collected in clean and autoclaved Nalgene (Nalge Europe, Hereford, UK) sampling bottles (volume 4.2 l), stored in dark cooling boxes at 4°C during transport and processed within 6 h after collection. For molecular biological analysis a known volume (usually 4.2 l) of spring water was filtered through polycarbonate membrane filters (Isopore™, 45 mm diameter, 0.2 μm pore size, Millipore, Bedford, USA). Immediately after filtration, filters were frozen and stored at −80°C until nucleic acid extraction. Nucleic acid extraction was performed as described by Griffiths and colleagues (2000), with a DNA precipitation using isopropanol instead of the polyethylene glycol. Recovered DNA was re-dissolved in 50 μl of sterile bi-distilled water and stored at −80°C until further analysis. All extracted sample DNAs were checked for amplifiable bacterial DNA and PCR inhibition by applying a general 16S rRNA gene PCR assay (Winter *et al*., 2007). Occasionally during Monitoring replicate samples (2 or 3 times 4.2 l) were taken and processed separately (*n* = 9). The sample replicates yielded very reproducible quantitative microbial source tracking results (e.g. the median coefficient of variation of BacR ME was 7%). The DNA from four samples (one each from May, April and September 2005 and one from January 2006) apparently was lost during PCR extraction and the respective sampling dates were excluded from all analysis.

Enumeration of *E. coli*, enterococci, presumptive *C. perfringens* and heterotrophic plate count at 22°C was performed as described in the respective ISO standard methods (ISO, 1999; 2000a,b; 2002). Numbers of aerobic spore-forming bacteria were determined by pasteurization of the water sample at 60°C for 15 min, membrane filtration and incubation on yeast extract agar at 22°C for 7 days.

### qPCR procedures

Human (BacH)- and ruminant (BacR)-specific qPCR assays were performed as described previously (Reischer *et al*., 2006; 2007). Shortly, qPCR was monitored on an iCycler iQ Real-Time Detection System (Bio-Rad, Hercules, USA). Reaction mixture composition for the BacH qPCR was (total volume of 25 μl): 2.5 μl of the respective sample DNA dilution, 200 nmol l^−1^ primer BacH_f, 200 nmol l^−1^ primer BacH_r, 100 nmol l^−1^ TaqMan MGB probe BacH_pC, 100 nmol l^−1^ TaqMan MGB probe BacH_pT, 10 μg of bovine serum albumin (Boehringer Ingelheim, Vienna, Austria), 12.5 μl of iQ Supermix (Bio-Rad); for the BacR qPCR (total volume of 25 μl): 2.5 μl of sample DNA dilution, 100 nmol l^−1^ primer BacR_f, 500 nmol l^−1^ BacR_r, 100 nmol l^−1^ TaqMan MGB probe BacR_p, 10 μg of bovine serum albumin (Boehringer Ingelheim), 12.5 μl of iQ Supermix (Bio-Rad), 2 mM additional MgCl_2_ (Bio-Rad). The PCR programme was the following: 95°C for 3 min, 50 cycles of 95°C for 15 s, 61°C (BacH) and 60°C (BacR), respectively, for 15 s and 72°C for 45 s. Real-time data were collected during the elongation step at 72°C. All reactions were performed in triplicates (three replicate reactions per sample dilution). All sample DNAs were measured in at least two 10-fold DNA dilution steps to rule out the presence of PCR inhibitory substances in the extracts. A total of six 10-fold serial dilutions of plasmid standard (10^0^−10^5^ gene copies) were run in triplicates on every well plate as well as a no-template control and a no-amplification control (containing plasmid standard and 0.01% sodium dodecyl sulfate). Quantitative microbial source tracking results were expressed as marker equivalents (ME) per litre taking account of the filtration volume and the DNA sample dilution yielding the result. A 4.2 l filtration volume, the use of 2.5 μl of undiluted DNA extract in qPCR and the minimal detectable marker concentration per reaction define the detection threshold (Reischer *et al*., 2006; 2007).

### Data processing and statistical analysis

Logarithmic transformations (log^+1^) were performed by calculation of log_10_ after addition of 1 to a given value. Statistical analyses were performed with the Statistical Package for the Social Sciences, version 14.0 (SPSS, Chicago, IL). For multiple testing, a Bonferroni correction was applied. Graphs were prepared using Sigma Plot 2001 (SPSS). Spearman rank correlation analysis included all samples regardless whether the quantitative microbial source tracking markers were detectable or not. Quantitative microbial source tracking results negative in qPCR were set to the detection threshold of 5 ME l^−1^. In order to assess the effect of including samples with no detactable quantitative microbial source tracking marker in the calculation, correlation analysis was repeated only with samples containing > 5 ME l^−1^ of both BacR and BacH. No significant change in the results for this reduced data set was observed.

### Sampling and DNA extraction of soil samples

Soil samples were collected on 10 and 11 July and 11 and 12 September 2006 in the LKAS2 catchment area in the Northern Calcareous Alps covering an area of approximately 70 km^2^ (for description of study area see Farnleitner *et al*., 2005). The altitude of the sampling sites ranged from 1200 to 2200 m a.s.l. Eighty-two soil samples were randomly collected throughout the study area on two walking tours. In addition soil samples from three type regions defined by vegetation type were collected: alpine pastures with and without livestock (47°38.9′N, 15°16.1′E, altitude 1500 m a.s.l.); pine woodland (47°38.6′N, 15°09.9′E, altitude 1300 m a.s.l.); and alpine swards above the tree line (47°37.6′N, 15°08.6′E, altitude 1990 m a.s.l.). At each sublocation (total *n* = 12) 10 individual soil samples were drawn out by a drill. Soil samples from two different depths (0–5 cm, 5–10 cm) were retrieved; samples from corresponding depths were pooled. In total 48 soil samples were collected. Soil samples were transported at 4°C, sieved (pore size < 2 mm) and stored at −20°C until DNA extraction. DNA was extracted from 250 mg of each soil sample using the Ultra Clean Soil DNA kit (MoBio Laboratories, Carlsbad, USA) in combination with bead-beating (FastPrep FP120, Bio-101, Vista, USA) following the manufacturer's recommendations. Cell lysis was performed at a machine speed setting of 6 for 30 s. DNA was stored at −20°C. All soil DNA extracts were measured in qPCR in the 10^−2^ dilution. This dilution was found not to inhibit qPCR by standard addition experiments.
